# Comparison of clinical performance between trifocal and bifocal intraocular lenses: A meta-analysis

**DOI:** 10.1371/journal.pone.0186522

**Published:** 2017-10-26

**Authors:** Zequan Xu, Danmin Cao, Xu Chen, Song Wu, Xin Wang, Qiang Wu

**Affiliations:** 1 Department of Ophthalmology, Shanghai Jiao Tong University Affiliated Sixth People's Hospital, No. 600, Xuhui District, Shanghai, PR China; 2 Aier School of Ophthalmology, Central South University, Tianxin District, Changsha, Hunan Province, PR China; 3 Department of Cataract and Glaucoma, Shanghai Aier Eye Hospital No. 1286, Shanghai, China, PR China; 4 School of Integrated Traditional and Western Medicine, Anhui University of Chinese Medicine, No. 103, Hefei, Anhui, PR China; 5 School of Data Science, Fudan University, No. 220, Yangpu District, Shanghai, PR China; Wenzhou Medical University Eye Hospital, CHINA

## Abstract

**Purpose:**

To compare the clinical performance between trifocal and bifocal intraocular lenses in bilateral cataract and/or refractive lens exchange (RLE) surgery.

**Methods:**

A comprehensive literature search of PubMed, EMBASE, Cochrane Controlled Trials Register and Web of Science was performed through October 2016 to identify randomized, controlled trials (RCTs) and comparative cohort studies. The primary outcomes were uncorrected distance visual acuity (UDVA), uncorrected intermediate visual acuity (UIVA), uncorrected near visual acuity (UNVA), defocus curve, spectacle independence, patient satisfaction and contrast sensitivity. The secondary outcomes were residual sphere, spherical equivalent (SE), cylinder and complications.

**Results:**

Six RCTs and 2 cohort studies including 568 eyes (278 in the trifocal group and 290 in the bifocal group) were identified. There was a statically significant difference between the two groups in UDVA (WMD: -0.03, 95% CI: -0.05 to -0.01, P = 0.005), but the difference (0.03 log MAR) is not clinically significant. Intermediate visual acuity was better in the trifocal IOL group judging from UIVA and defocus curves. There was a statically significant difference between the two groups in residual cylinder (WMD: 0.11, 95% CI: 0.02 to 0.20, P = 0.02), and subgroup AT Lisa tri 839MP trifocal also showed significant better UNVA than bifocal IOLs (WMD: -0.13, 95% CI: -0.17 to -0.08, P<0.00001). However, no significant differences were observed in UNVA (WMD: -0.04, 95% CI: -0.11 to 0.02, P = 0.19), spectacle independence (WMD: 1.27, 95% CI: 0.89 to 18.15, P = 0.07), patient satisfaction (WMD: 4.01, 95% CI: 0.07 to 22.72, P = 0.87), residual sphere (WMD: -0.03, 95% CI: -0.18 to 0.13, P = 0.74), SE (WMD: 0.04, 95% CI: -0.09 to 0.16, P = 0.55) or complications (WMD: 2.08, 95% CI: 0.35 to 12.43, P = 0.42).

**Conclusions:**

Trifocal IOL technology (especially AT Lisa trifocal 839M trifocal) had a clear advantage over bifocal IOLs in intermediate visual acuity, while both trifocal IOLs and bifocal IOLs showed excellent performance in distance visual acuity. AT Lisa trifocal 839M trifocal could provide better uncorrected near visual acuity than bifocal IOLs. However, more evidence is needed to compare their spectacle independence, higher satisfaction rate, and photic phenomena.

## Introduction

The gradual loss of lens accommodation as a person ages (resulting in presbyopia) or as a result of a surgical procedure (mainly cataract surgery) is a reason why patients seek treatment.[1] A range of surgical procedures are available to restore the accommodation, such as application of laser and corneal inlays[2,3], the implantation of accommodating intraocular lenses (IOLs)[4], etc. Among these procedures, multifocal IOLs constitute the first choice [5–8] for many surgeons due to their ability to provide functional uncorrected vision over a range of distances, and spectacle independence is expected to be achieved. However, multifocal IOLs (both bifocal and trifocal IOL) are still associated with some drawbacks. For one aspect, with traditional bifocal IOLs, the intermediate distance range is still penalized, compared with the far and near distance ranges; for another aspect, because multifocal IOL designs divide the incoming light into more than one focus, the effect of the light in out-of-focus images reduces the contrast of in-focus images. This effect also reduces contrast sensitivity and modulation transfer function (MTF) [5], and unwanted visual phenomena, including glare and halos^[^^9^^]^, can occur.

The trifocal IOL is a new type of multifocal IOL designed to supply better intermediate visual acuity without impairing near and far vision.^10^ The first trifocal IOL was the Fine Vision Micro F IOL (PhysIOL, Liège, Belgium), it has 21.0 D base power, +3.33 D near add power, and +1.66 D intermediate add power [10]. Many studies have analyzed the clinical performance [10–18] and experimental performance [19–22] of this IOL. Another trifocal IOL is the AT Lisa tri 839MP (Carl Zeiss Meditec, Germany); it has 20.0 D base power, +3.5 D near add power, and +1.75 D intermediate add power. Many researchers have also studied its clinical performance[23–36] and experimental performance[22,37,38]. Another new trifocal IOL, the Pan Optix Presbyopia Correcting IOL (Alcon Research, Fort Worth, TX, USA)[39], still lacks a comparative or cohort study. Most studies of these trifocal IOLs have obtained encouraging results in clinical performance.

One question has inevitably arisen with the advent of trifocal IOLs: whether two foci, distance and near, adequately address visual needs or whether an intermediate foci is required.[5,40] It should be noted that an added intermediate focus results in better intermediate VA, but it also causes two permanent defocus images instead of one [20], as well as greater complexity of the manufacturing process, both of which could degrade the optical quality of the lens.

The answer to these questions mostly relies on which type of IOL obtains better visual acuity, satisfaction of patients and spectacle independence. The answer, however, has not always been consistent; thus, we conducted a systematic review and meta-analysis of the published randomized, controlled trials and cohort comparison studies to compare the clinical performance metrics mentioned above following bilateral implantation of trifocal IOLs and bifocal IOLs after cataract and/or refractive lens exchange (RLE) surgery.

## Sources and methods

This meta-analysis was reported in accordance with the Preferred Reporting Items for Systematic Reviews and Meta-Analysis (PRISMA) statement [41,42] and was registered at the International Prospective Register of Systematic Reviews (number CRD 42016048566).

### Search strategy

To conduct a systematic search, PubMed, EMBASE, the Cochrane Controlled Trials Register and Web of Science were searched for articles dated through October 2016. We used the following combined text and MeSH terms: (((((trifocal[Title/Abstract]) AND ((((("Lenses, Intraocular"[Mesh]) OR "Phakic Intraocular Lenses"[Mesh])) OR intraocular lens [Title/Abstract]) ORIOL[Title/Abstract]))) OR Panoptix [Title/Abstract]) OR at lisa tri[Title/Abstract]) OR finevision [Title/Abstract]. No restrictions were placed on the language of publication. In addition, for potential trials that might have been missed in the primary searches, we also performed a manual search.

### Inclusion and exclusion criteria

The inclusion criteria were studies that reported the clinical outcomes of trials implanting trifocal IOLs and bifocal IOLs involving patients who underwent cataract and/or RLE surgery.

The exclusion criteria were studies involving patients with coexisting pathology, amblyopia, or previous IOL implantation or laser refractive surgery. If identified, studies reporting double data were excluded to retain only one.

### Screening process

Two independent reviewers (Ze-quan Xu and Dan-min Cao) respectively conducted a preliminary review of the titles and abstracts. Subsequently, the full articles were carefully analyzed to select the studies that met the criteria mentioned above.

Disagreement between Xu and Cao was resolved through careful discussion—resorting to a third reviewer (Xu Chen) when necessary—until a consensus was reached.

### Quality assessment

The Cochrane Collaboration’s tool for risk of bias [43] was used to evaluate the quality of RCTs. In short, all of the parameters (including sequence generation, allocation concealment, etc.) were graded as having a low risk of bias, high risk of bias, or unclear risk of bias.

For the included cohort studies, the Newcastle-Ottawa Scale (NOS)[44] was used for quality assessment. The NOS requires a total of nine stars, and a score of at least 7 stars indicates good quality. On this scale, a total of four stars are given for patient selection, three for outcome assessment and two for comparability.

### Data collection

The available data included study design, IOLs implanted, whether the study group underwent cataract or RLE surgery, the number of patients, UDVA, UIVA and UNVA, defocus curve, spectacle independence, patient satisfaction, contrast sensitivity, residual sphere, SE and cylinder, and postoperative complications.

### Outcome measurements

Binocular UDVA, UIVA and UNVA were recorded in the following manner: using a 100% contrast chart and without spectacles, UDVA was recorded at 4 or 6 m, UIVA at 70 or 63 or 66 cm and UNVA at 40 or 33 cm. All of the VA values were recorded as logMAR value, while UDVA, UIVA and UNVA could also be demonstrated in defocus curves.

Spectacle independence was described as having functional vision in different circumstances and at different distances with no use of spectacles.

Patient satisfaction and photic phenomena (side effects) were reported by a validated questionnaire, such as the NEI-RQL, NEI VFQ, and VF-14.

Contrast sensitivity testing was performed under photoptic conditions with luminance of 85 cd/m^2^, as well as mesoptic conditions with luminance of 3 cd/m^2^.

In addition, data on the postoperation residual sphere, SE, cylinder, and postoperation complications were also collected if provided.

### Statistical analysis

All of the statistical analyses were performed using Rev Manager Software (version 5.3; Cochrane Collaboration, Oxford, United Kingdom). Statistical heterogeneity was tested using the chi-square and I^2^ statistic. When showing significant heterogeneity (I^2^>50%), a random-effects meta-analysis was used; otherwise, fixed-effects models were used. [45]. The weighted mean difference (WMD) with a 95% CI was calculated. A p-value of less than 0.05 was considered statistically significant.

## Results

### Literature search

**Fig 1:**
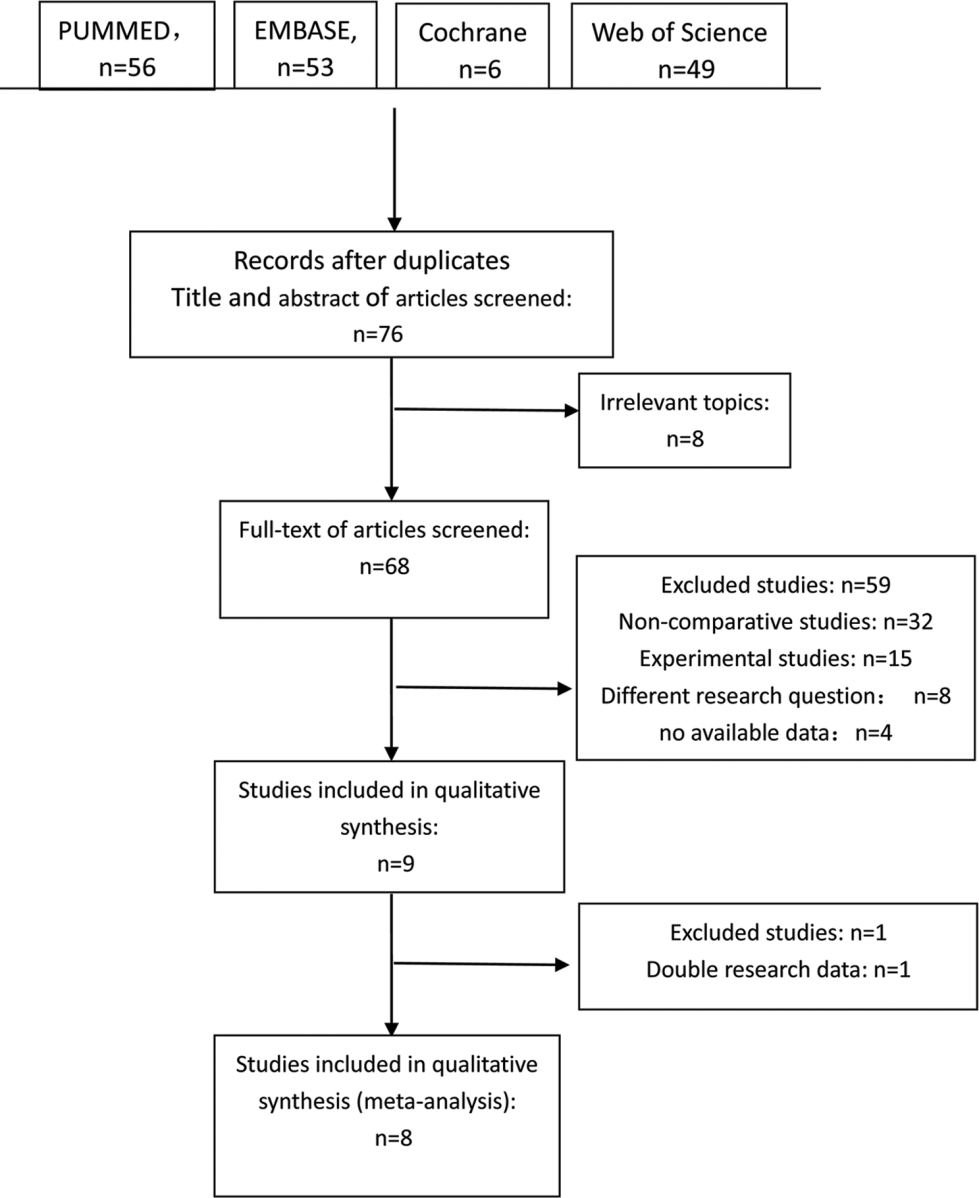
Flow diagram of the literature search in this meta-analysis.

The search identified a total of 76 abstracts after duplicates, of which 68 met the above inclusion criteria. Full versions of all 68 papers were scrutinized against the exclusion criteria; 59 papers were excluded for the following reasons: non-comparative studies, n = 32; experimental studies, n = 15; different research question = 8; and no available data: n = 4. Of the nine studies included in the qualitative synthesis, one study was excluded because this study and another study[46,47] reported double data. Finally, there were eight studies[33,47–53] included in the qualitative meta-analysis. The trial selection process is shown in Fig 1. There were two totally different studies both performed by Gundersen in 2016 and two studies performed by Plaza in 2016, and part of the data (the data on trifocal IOL groups) from the two studies are duplicated.

### Risk of bias assessment

None of the included RCTs[48–53] described the specific methods of random sequence generation or blinding of outcome assessments, except for one RCT that mentioned the blinding of patients [48], and only one study performed randomization using the software provided by www.random.org.[50] Actually, in an IOL exchange surgery, masking of the surgeon is impossible, and persuading the patients to randomly choose between trifocal and bifocal IOLs, which differentiate sharply in price, is difficult. Thus, randomization is quite difficult to realize in these studies. After discussions of our team, we finally downgraded these RCTs to non-randomized comparative studies in our meta-analysis, and their quality was assessed by the NOS as in other cohort studies [33,46], as shown in Table 1. All of the studies described missing patients, but no study had missing cases, and all the of studies reported all of their main results; thus, all of them had two stars for comparability (two stars maximum) and three stars for outcome assessment (three stars maximum). However, some studies had flaws in patient selection (four stars maximum): one study did not match preoperative near visual acuity, [48] one study did not match preoperative uncorrected visual acuity, [33] one study did not match preoperative corrected visual acuity, [51] and three studies did not discuss preoperative VA at all. [49,52,53]

**Table 1:** Characteristics of studies (n = 8) included in the meta-analysis.

Study	Site	Design	Procedure	Patients: trifocal / bifocal	Trifocal IOL	Bifocal IOL	Scores on Newcastle-Ottawa Scale	Follow-up
Jonker et al. (2015)	The Netherlands	R	Cataract	15/13	Fine vision (NA = +3.5 D, IA = +1.75 D)	ReSTOR SN6AD1 (NA = +3 D)	Patient selection: 2 Comparability: 2 outcome assessment: 3	6 m
Gundersen et al. (2016)1	Norway	R	Cataract	11/11	Fine vision toric (NA = +3.5 D, IA = +1.75 D)	ReSTOR SND1T (NA = +3 D)	Patient selection: 3 Comparability: 2 outcome assessment: 3	3 m
Cochener et al. (2016)	France	R	Cataract	15/12	Fine vision (NA = +3.5 D, IA = +1.75 D)	Tecnis ZMB00 (NA = +4 D)	Patient selection: 4 Comparability: 2 outcome assessment: 3	1–6 m
Brito et al. (2015)	Portugal	C	RLE	16/8	Lisa tri 839M (NA = +3.33 D, IA = +1.66 D)	Lisa 909MP (NA = +3.75 D)	Patient selection: 2 Comparability: 2 outcome assessment: 3	7–10 m
Mojzis et al. (2014)	Czech Republic	R	RLE/cataract	15/15	Lisa tri 839MP (NA = +3.33 D, IA = +1.66 D)	Lisa 801 (NA = +3.75 D)	Patient selection: 3 Comparability: 2 outcome assessment: 3	3 m
Gundersen et al. (2016)2	Norway	R	RLE/cataract	25/30	Lisa tri 839MP (NA = +3.33 D, IA = +1.66 D)	ReSTOR SN6AD1/ SN6AD2 (NA = +3/+2.5 D)	Patient selection: 3 Comparability: 2 outcome assessment: 3	3–12 m
Plaza et al.(2016)1, 2	Spain	C	Cataract	30/45	Lisa tri 839MP (NA = +3.33 D, IA = +1.66 D)/ Fine vision (NA = +3.5 D, IA = +1.75 D)	ReSTOR SN6AD1 (NA = +3 D)/Mplus-LS31 (NA = +3 D)Acri.Lisa 366 D (NA = +3.75 D)	Patient selection: 4 Comparability: 2 outcome assessment: 3	3 m
Bilbao et al. (2016)	Spain	R	RLE/cataract	12/11	Fine vision (NA = +3.5 D, IA = +1.75 D)	ReSTOR SN6AD1 / SN6AD2 (NA = +3/+2.5 D)	Patient selection: 3 Comparability: 2 outcome assessment: 3	3 m

R = randomized, controlled trial (RCT); C = comparative cohort trial; RLE = refractive lens change; NA = near add; ID = intermediate add. There were totally different two studies both performed by Gundersen in 2016 and two studies performed by Plaza in 2016; part of the data (the data of the trifocal IOL group) from the two studies is duplicated.

### Characteristics of included studies

In the present meta-analysis, Table 1 shows the characteristics of the 8 studies, and Table 2 shows the summary of outcomes (including overall quality of evidence judging from GRADE/GDT) in the meta-analysis.

**Table 2:** Summary of outcomes included in the meta-analysis.

Outcome	Risk for trifocal IOL	No. of Participants (studies)	Importance	Quality	Comments
**UDVA**	The intervention group was 0.03 lower (0.05 to 0.01 lower)	206 (6 studies)	CRITICAL	⊕⊕⊕⊕high^1, 2^	The difference is not clinically significant
**UIVA**	The intervention group was 0.07 lower (0.2 lower to 0.05 higher)	85 (3 studies)	CRITICAL	⊕⊝⊝⊝ very low^3^	Also reflected by VA at -1.5 D on defocus curve
**UNVA**	The intervention group was 0.04 lower (0.11 lower to 0.02 higher)	184 (5 studies)	CRITICAL	⊕⊕⊝⊝ low^1, 2^	See subgroup analysis in Fig 3
**spectacle independence**	Relative effect (95% CI): OR 4.01 (0.89 to 18.15)	54 (2 studies)	CRITICAL	⊕⊕⊕⊝ moderate^2^	I^2^ = 0
**Satisfaction**	Relative effect (95% CI): OR 1.27 (0.07 to 22.72)	49 (2 studies)	CRITICAL	⊕⊕⊕⊝ moderate^2^	Heterogeneity is not applicable because of the ceiling effect
**Postoperative complications**	Relative effect (95% CI): OR 2.08 (0.35 to 12.43)	78 (3 studies)	IMPORTANT	⊕⊕⊕⊝ moderate^2^	I^2^ = 0
**Residual phere**	The intervention group was 0.02 lower (0.18 lower to 0.13 higher)	156 (4 studies)	IMPORTANT	⊕⊕⊕⊝ moderate^2^	I^2^ = 0
**Residual SE**	The intervention group was 0.05 higher (0.07 lower to 0.16 higher)	185 (5 studies)	IMPORTANT	⊕⊕⊕⊕ high^1, 2^	I^2^ = 0
**Residual cylinder**	The intervention group was 0.11 higher (0.02 to 0.2 higher)	271 (7 studies)	IMPORTANT	⊕⊕⊕⊕high^1, 2^	I^2^ = 0

UDVA = uncorrected distance visual acuity; UIVA = uncorrected intermediate visual acuity; UNVA = uncorrected near visual acuity; SE = spherical equivalent refraction. Visual symptoms included halo, glare and others. CI: Confidence interval; OR: Odds ratio; 1 large sample; 2 no confounding factors could change the effect; 3 the results of uncorrected intermediate visual acuity were not consistent with defocus curves

### Primary outcomes

The primary outcomes were UDVA, UIVA, UNVA, defocus curve, spectacle independence, patient satisfaction, contrast sensitivity and phenomena (side effects)

#### Uncorrected distance visual acuity (UDVA)

**Fig 2:**
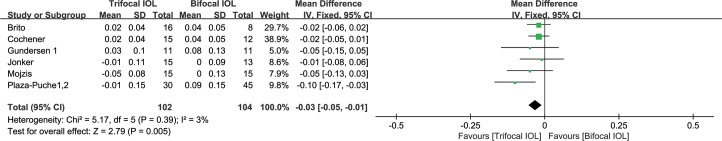
Meta-analysis of postoperative binocular uncorrected distance visual acuity (UDVA). SD = standard deviation; CI = confidence interval.

There were six studies [33,46,48–51] reporting UDVA. These studies had a heterogeneity effect size of I^2^ = 3% (P < 0.00001). Thus, the fixed-effect model was used. The mean UDVA in the trifocal group was significantly better than that in the bifocal group (WMD: -0.03, 95% CI: -0.05 to -0.01, P = 0.005) (Fig 2). Although the difference was significantly different, 0.03 logMAR was not clinically significant (0.1 logMAR was to be assumed clinically significant [53]), and in Table 3, there was no difference in distance visual acuity (0.00 D, corresponding to distance vision). The quality of the evidence was high (in S1 Fig)

**Table 3:** Comparison of defocus curve between trifocal and bifocal IOL.

Study	Trifocal IOL	Bifocal IOL	Trifocal IOL got better performance	Bifocal IOL got better performance
**Jonker et al. (2015)**	**Fine vision**	**ReSTOR SN6AD1**	**Significant differences were found in -1 D; insignificant differences were found in 2 D, 1.5 D, 1 D, 0.5 D, 0 D, -0.5 D, -1.5 D, -2.5 D, -3 D**	**Insignificant differences were found in -2 D, -3.5 D, -4 D**
**Gundersen et al. (2016)1**	**Fine vision**	**ReSTOR SND1T**	**Significant differences were found in 2 D, -1.5 D; insignificant differences were found in 1.5 D, 1 D, 0 D, -0.5 D, -1 D, -2 D, -2.5 D, -3 D, -3.5 D, -4 D**	**Insignificant differences were found in 0.5 D**
**Cochener et al. (2016)**	**Fine vision**	**Tecnis ZMB00**	**Significant differences were found in -1 D, -1.5 D, -2 D, -2.5 D; insignificant differences were found in0 D, -0.5 D, -3 D, -3.5 D**	**Insignificant differences were found in 2 D, 1.5 D, 1 D, 0.5 D**
**Bilbao et al. (2016)**	**Fine vision**	**ReSTOR SN6AD1 / SN6AD2**	**Significant differences were found in -1.5 D, -2 D, -2.5 D, -3 D, -3.5 D; insignificant differences were found in 2 D, 1.5 D, 1 D, 0.5 D, 0 D, -0.5 D, -1 D**	Not found
**Plaza et al. (2016)1, 2**	**Fine vision**	**ReSTOR SN6AD1 / Mplus-LS31 / Acri.Lisa 366 D**	**Significant differences were found in -1 D, -1.5 D, -2 D, -2.5 D, -3 D, -3.5 D, -4 D; insignificant differences were found in 1 D, 0.5 D, 0 D, -0.5 D**	Not found
**Mojzis et al. (2014)**	**Lisa tri 839MP**	**Lisa 801**	**Significant differences were found in -1 D, -1.5 D; insignificant differences were found in 0 D, -0.5 D, -2 D, -2.5 D**	**Significant differences were found in -3.5 D, -4 D; insignificant differences were found in 1 D, 0.5 D, -3 D**
**Gundersen et al. 2 (2016)**	**Lisa tri 839MP**	**ReSTOR SN6AD1/ SN6AD2**	**Significant differences were found in -0.5 D, -1 D, -1.5 D; insignificant differences were found in 1 D, 0.5 D**	**Insignificant differences were found in 0 D, -2 D**
**Plaza et al. 1, 2 (2016)**	**Lisa tri 839MP**	**ReSTOR SN6AD1/Mplus-LS31/Acri.Lisa 366 D**	**Significant differences were found in -0.5 D, -1 D, -1.5 D, -2 D, -2.5 D, -3 D, -3, 5 D, -4 D; insignificant differences were found in 1 D, 0.5 D, 0 D**	Not found

#### Uncorrected intermediate visual acuity (UIVA)

There were only three studies[48,50,51] reporting UDVA as the mean ± SD using different methods. These studies had a heterogeneity effect size of I^2^ = 88% (P < 0.0002). Thus, the random-effect model was used. The mean UIVA in the trifocal group was insignificantly better than that in the bifocal group (WMD: -0.07, 95% CI: -0.20 to 0.05, P = 0.25). The quality of the evidence was very low (in S3 Fig). However, the AT Lisa tri 839MP trifocal (the study by Mojzis) had significantly (both statistically and clinically) better UIVA than bifocal IOLs (WMD: -0.23, 95% CI: -0.33 to -0.13) (S2 Fig).

At the same time, seven studies[46,48–53] reported VA at -1.5 D at the defocus curve (Table 3). Of these seven studies, four studies[48–50,53] involved Fine Vision trifocal IOLs, two[51,52] involved AT Lisa tri 839MP trifocal IOLs, and one study[46] involved both the Fine Vision trifocal IOL and AT Lisa tri 839MP trifocal IOL. Three studies indicated that the AT Lisa tri 839MP trifocal IOL showed significantly better UIVA than bifocal IOLs, and four studies indicated that Fine Vision trifocal IOLs had significantly better UIVA than bifocal IOLs.

#### Uncorrected near visual acuity (UNVA)

**Fig 3:**
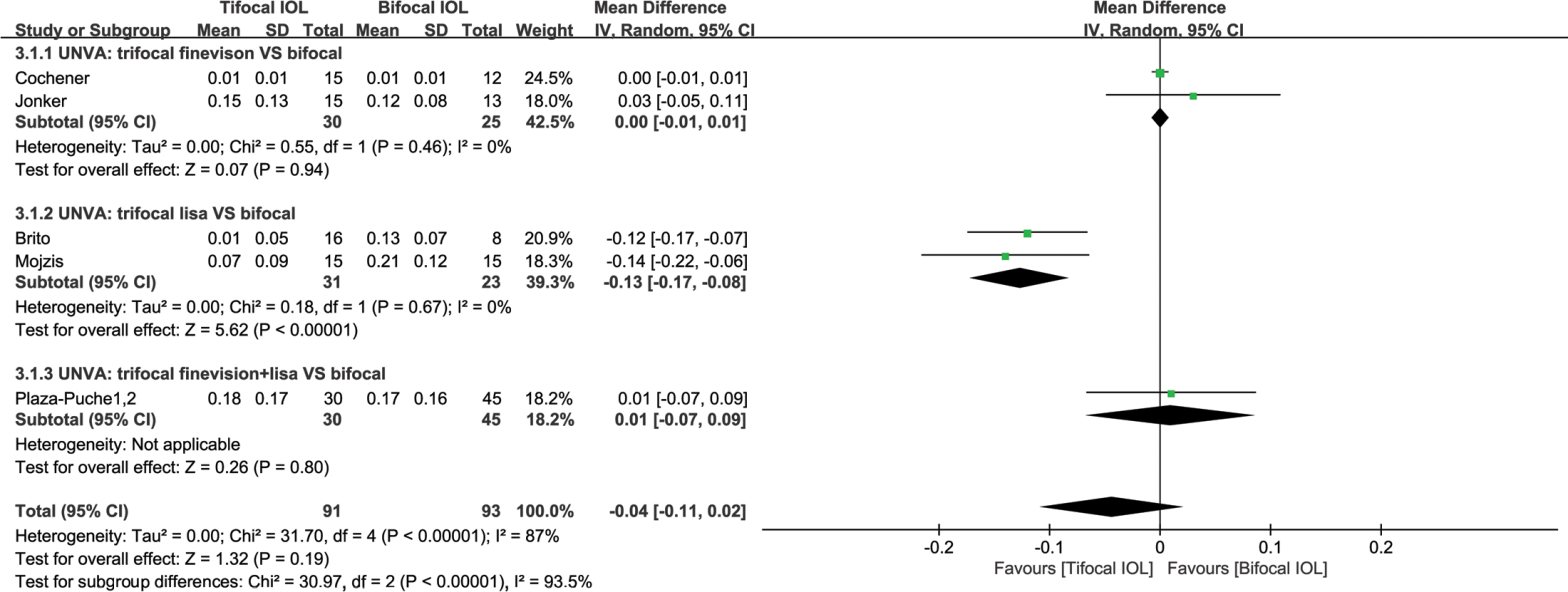
Meta-analysis of postoperative binocular uncorrected near visual acuity (UNVA). SD = standard deviation; CI = confidence interval; fine vison = Fine Vision Micro F IOL; lisa = AT Lisa tri 839MP IOL.

There were five studies [33,46,48,50,51,53] reporting UNVA. These studies had a heterogeneity effect size of I^2^ = 87% (P < 0.00001). Thus, the random-effect model was used. The mean UNVA in the trifocal group was insignificantly better than that in the bifocal group (WMD: -0.04, 95% CI: -0.11 to 0.02, P = 0.19) (Fig 3). The quality of the evidence was low (in S4 Fig).

Subgroup analysis according to the type of trifocal IOL involved was also conducted. These studies were divided into three subgroups: Fine Vision (subgroup 1); AT Lisa tri 839MP trifocal (subgroup 2); and mixed Fine Vision and AT Lisa tri 839MP trifocal (subgroup 3). The results from subgroup 2 revealed that the AT Lisa tri 839MP trifocal obtained significantly (both statistically and clinically) better UNVA than bifocal IOLs (WMD: -0.13, 95% CI: -0.17 to -0.08, P<0.00001) (Fig 3).

#### Defocus curve

There were seven studies [45, 47–52] reporting defocus curves (Table 3). One study (Plaza et al, 2016)[46] reported on both the trifocal IOL subgroups (Fine vision group and AT Lisa tri 839MP group), so we compared the trifocal IOLs in the two subgroups with the bifocal group separately, and the general results showed that trifocal IOLs had better performance from -2.5 D to 0 D, and the AT Lisa tri 839MP group had significantly better performance from -0.5 D to -1.5 D.

#### Spectacle independence

**Fig 4:**
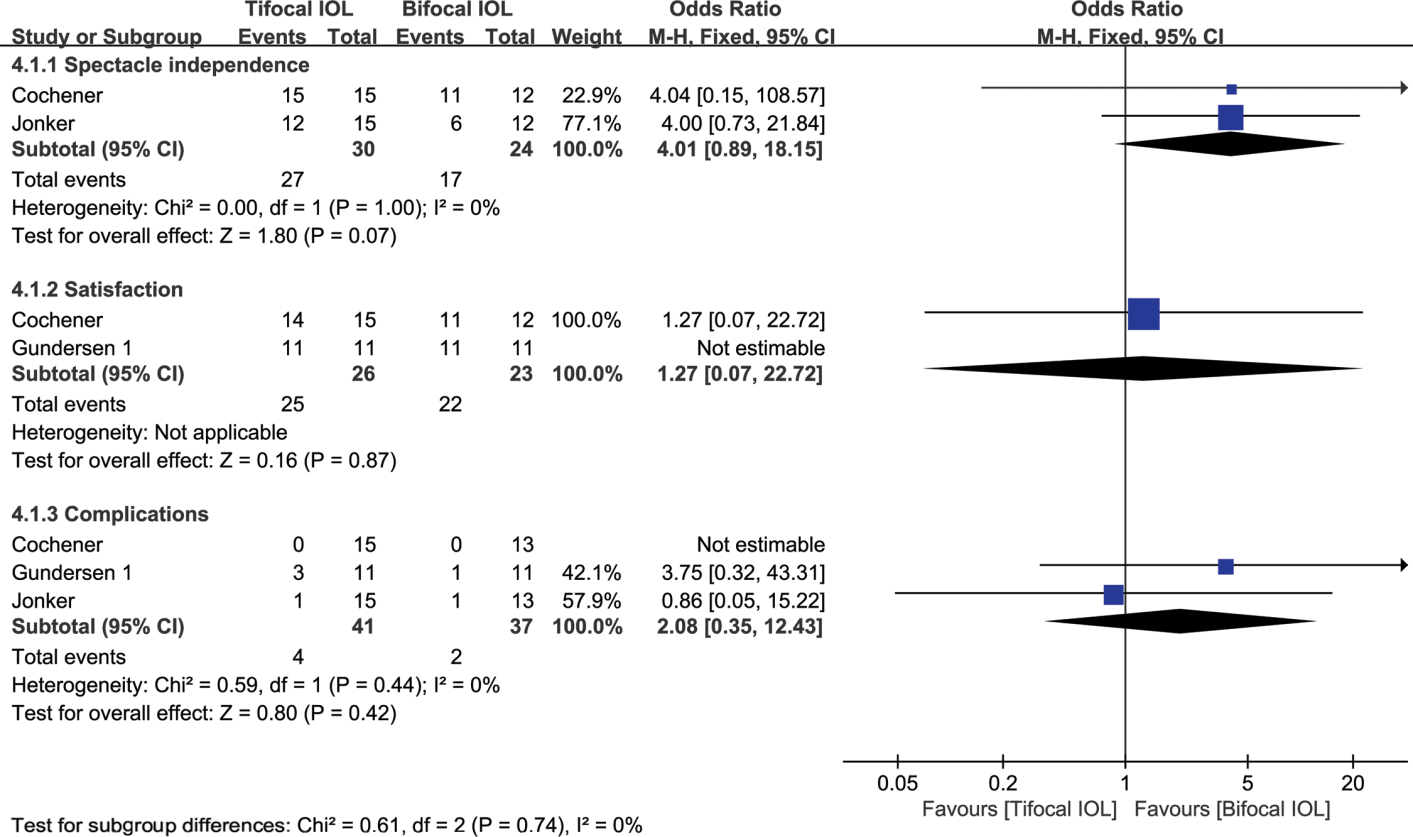
Meta-analysis of postoperative spectacle independence, patient satisfaction and complications. SD = standard deviation; CI = confidence interval.

There were only two studies [48,50] reporting spectacle independence. The spectacle independence in the trifocal group was insignificantly better than that in the bifocal group (WMD: 1.27, 95% CI: 0.89 to 18.15, P = 0.07) (Fig 4). The quality of the evidence was moderate (in S5 Fig)

#### Patient satisfaction

There were only two studies[49,50] reporting spectacle independence. The spectacle independence in the trifocal group was insignificantly better than that in the bifocal group (WMD: 4.01, 95% CI: 0.07 to 22.72, P = 0.87) (Fig 4). The quality of the evidence was moderate (in S5 Fig)

#### Contrast sensitivity

There were 4 studies[48,50,51,53] reporting contrast sensitivity under photoptic conditions (Table 4); contrast sensitivity was insignificantly better at 6 c/d and 12 c/d in the trifocal group, and there was a study reporting contrast sensitivity under mesoptic conditions[48], with contrast sensitivity significantly worse at 6 c/d in the trifocal group. There was also a study reporting contrast sensitivity under both photoptic and glare conditions[50], and contrast sensitivity was insignificantly better at 6 c/d and 12 c/d and insignificantly worse at 1.5 c/d in the trifocal group.

**Table 4:** Comparison of contrast sensitivity between trifocal and bifocal IOL under photoptic conditions.

Study	Trifocal IOL	Bifocal IOL	Trifocal IOL showed better performance	Bifocal IOL showed better performance
**Jonker et al.** **(2015)**	Fine vision	ReSTOR SN6AD1	Insignificant differences were found in 3, 6, 12 c/d	Not found
**Cochener et al.** **(2016)**	Fine vision	Tecnis ZMB00	Insignificant differences were found in 6, 12 c/d	Insignificant differences were found in 1.5, 3 c/d
**Bilbao et al.** **(2016)**	Fine vision	ReSTOR SN6AD1 / SN6AD2	Significant differences were found in 3 c/d; insignificant differences were found in 1.5, 6, 12, 18 c/d	Not found
**Mojzis et al.** **(2014)**	Lisa tri 839MP	Lisa 801	Insignificant differences were found in 6, 12, 18 c/d	Insignificant differences were found 3 c/d

#### Phenomena (side effects)

The occurrence of side effects with multifocal IOLs, mainly glare and halos, was also reported in two studies. For glare, Jonker reported[48] a score of 55 in the trifocal group and 61 in the bifocal group on the NEI-RQL questionnaire (a score of 100 refers to the best quality of life); however, Cochener[50] reported that 92% of patients suffered from glare in the trifocal group, compared to only 67% in the bifocal group. Cochener[50] also reported that 58% of patients suffered from halo in the trifocal group, compared to only 50% in the bifocal group.

### Secondary outcomes

The secondary outcomes were residual sphere, spherical equivalent (SE) and cylinder, and post-operative complications.

#### Residual sphere

**Fig 5:**
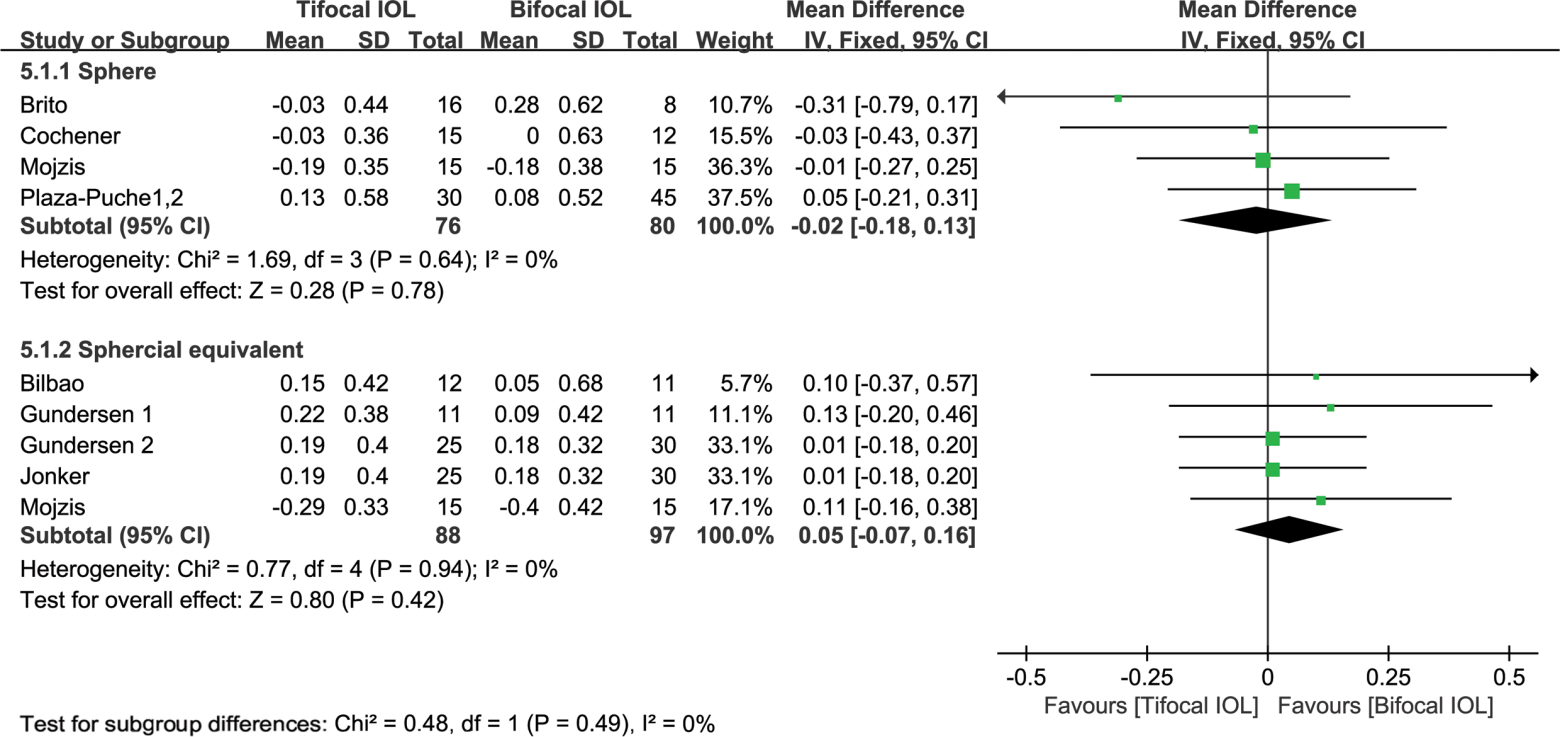
Meta-analysis of postoperative residual sphere and spherical equivalent (SE). SD = standard deviation; CI = confidence interval.

There were four studies[33,46,50,51] reporting residual sphere. These studies had a heterogeneity effect size of I^2^ = 0 (P = 0.66). Thus, the fixed-effect model was used. There was no significant difference between the two groups (WMD: -0.03, 95% CI: -0.18 to 0.13, P = 0.78) (Fig 5). The quality of the evidence was moderate (in S6 Fig).

#### Residual SE

There were five studies[48,49,51–53] reporting residual SE. These studies had a heterogeneity effect size of I^2^ = 0 (P = 0.96). Thus, the fixed-effect model was used. There was no significant difference between the two groups (WMD: 0.04, 95% CI: -0.09 to 0.16, P = 0.55) (Fig 5). The quality of the evidence was high (in S6 Fig).

#### Residual cylinder

**Fig 6:**
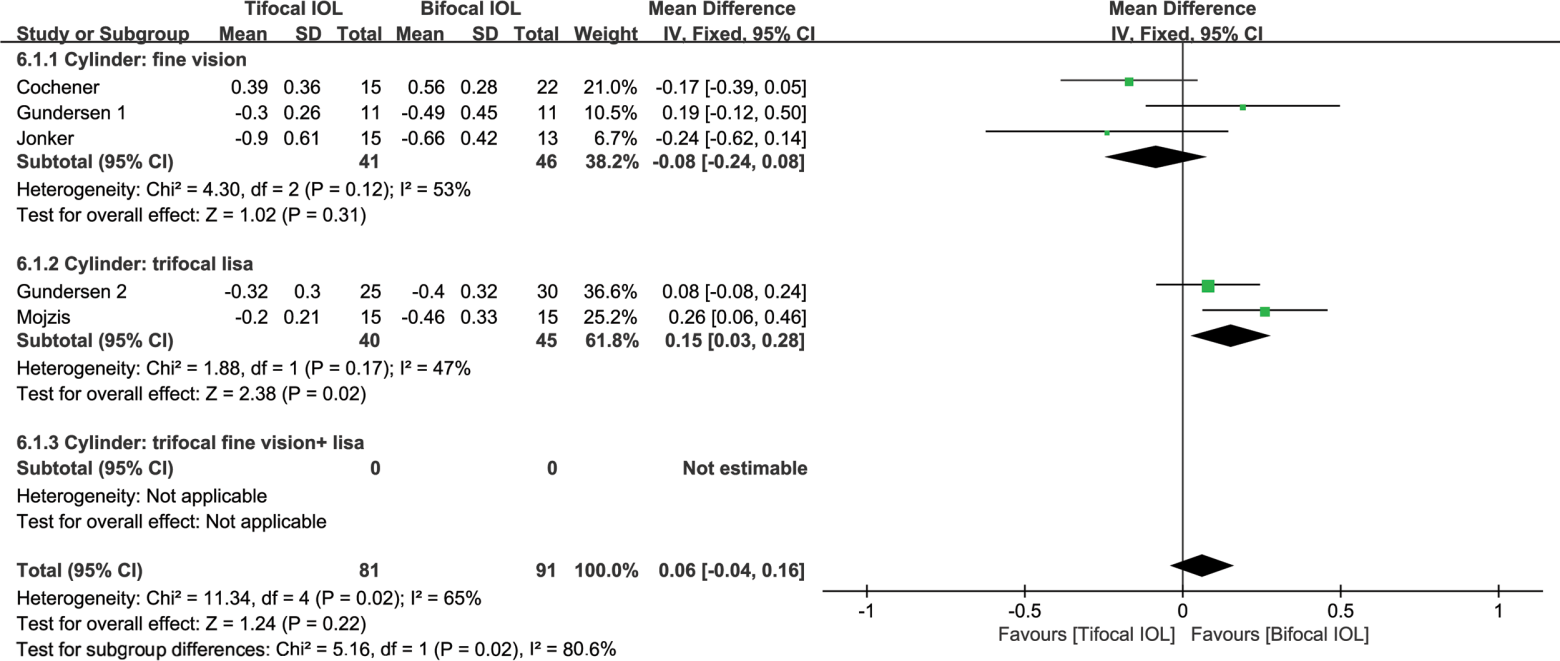
Meta-analysis of postoperative residual cylinder. SD = standard deviation; CI = confidence interval; fine vison = Fine Vision Micro F IOL; lisa = AT Lisa tri 839MP IOL.

There were seven studies[33,46,48–52] reporting residual cylinder. These studies had a heterogeneity effect size of I^2^ = 18% (P = 0.29). Thus, the fixed-effect model was used. There was no significant difference between the two groups (WMD: 0.11, 95% CI: 0.02 to 0.20, P = 0.02) (Fig 6). The quality of the evidence was moderate (in S7 Fig)

Subgroup analysis according to the type of trifocal IOL was also conducted. The studies were divided into three subgroups: Fine Vision (subgroup 1); AT Lisa tri 839MP trifocal (subgroup 2); and mixed Fine Vision and AT Lisa tri 839MP trifocal (subgroup 3). The results from subgroup 2 revealed that the AT Lisa tri 839MP trifocal had significant higher residual cylinder (absolute value) than bifocal IOLs (WMD: 0.13, 95% CI: 0.01 to 0.25, P = 0.03) (Fig 6); however, a higher cylinder indicates a lower absolute value since the value of the cylinder is less than zero.

#### Post-operative complications

There were only three studies[48–50] reporting complications (decentration>5°). These studies had a heterogeneity effect size of I^2^ = 0 (P = 0.44). Thus, the fixed-effect model was used. There was no significant difference between the two groups (WMD: 2.08, 95% CI: 0.35 to 12.43, P = 0.42) (Fig 4). The quality of the evidence was moderate (in S5 Fig).

## Discussion

To our knowledge, this study was the first meta-analysis to compare clinical performance between bifocal and trifocal IOLs, and it showed the realistic benefits of the recently developed trifocal IOL technology. As we mentioned above, the answer of the question of whether an intermediate focal IOL (the essence of the trifocal IOL) is required mostly relies on the visual acuity (far, near and intermediate) performance, satisfaction of patients and spectacle independence of the trifocal IOL.[5,40]

We found significantly better UDVA following trifocal IOL implants; however, the difference was not clinically significant. It was a very encouraging result since UDVA following bifocal IOL implants was already satisfying: the mean UDVA following bifocal IOL implants was 0.04±0.001 logMAR based on 37 studies of 6334 patients in a previous meta-analysis[7]; our counterpart was 0.057±0.129 logMAR following bifocal IOL implants, while it was -0.002±0.107 logMAR following trifocal IOL implants, based on 6 studies of 102 patients. To conclude, we are totally convinced that both trifocal and bifocal IOLs have excellent performance (approximately 0 logMAR) in distance visual acuity.

The demand for intermediate vision, which is important for daily activities at arm’s length or longer, is high for most patients, especially those younger than 65 years old[6]. There were only 3 studies reporting the mean value and SD of UIVA. To make it more complicated, different tests and variable distances (66 cm[50,51] or 70 cm[48]) were used for intermediate visual acuity measurements, and the evidence of the meta-analysis of the results was very low. However, binocular defocus curves of seven included studies showed that the trifocal group had statistically significantly better VA at -1.5 D (corresponding to the vision of a target at 67 cm) compared to the bifocal group, especially in the AT Lisa tri 839MP trifocal subgroup. It is worth noting that, in our meta-analysis, the AT Lisa tri 839MP trifocal had statistically significantly better UIVA than bifocal IOLs, and the difference was of clinical significance (0.23logMAR). Actually, better intermediate visual acuity is entirely expected with trifocal IOLs since the essence of a trifocal IOL is a true intermediate focal point. A few previous benchmark studies [20,21,38,54] have found that the trifocal lens provided a true third intermediate focal point not found with the bifocal lens. Further in Carson et al.’s study, [20] both the AT LISA tri and Fine Vision trifocal IOLs demonstrated an intermediate focal point at approximately 80 cm, while ReSTOR demonstrated no intermediate focal point. The intermediate-focus MTF values of the AT LISA tri and Fine Vision at 20/20 visual acuity were similar to each other (14.0% and 13.6% for the Fine Vision and AT LISA tri, respectively), while the counterpart values of the ReSTOR IOL were much lower (5.0% -7.2%). Similar results were also observed in other studies.[55] Thus, we are totally convinced that the introduction of a third focal point could effectively enhance the VA in the intermediate distance, especially with the AT Lisa tri 839MP trifocal IOL.

We found significantly better UNVA following trifocal IOL implantation in a subgroup (AT Lisa tri 839MP group) of implants than after bifocal implants, but no significant differences between the trifocal and bifocal groups were found in other subgroups. In Carson et al.’s study [20], the near foci of the AT Lisa tri 839MP were in the range of 38 to 40 cm, while the near foci of bifocal IOLs were in the range of 44–53 cm, which could partially explain why some trifocal IOLs had better UNVA performance since UNVA in this meta-analysis was measured at 33 cm[33,50] or 40 cm.[46,48,51]

What we found in the outcomes of defocus were consistent with UDVA, UIVA and UNVA measured with standard tests. A continuous defocus curve was found in trifocal IOLs: all of the bifocal IOLs had a V-pattern defocus curve with a sharp gap for intermediate vision, while trifocal IOLs had a minimal decrease in the VA at the intermediate range (at a vergence of -1.5 D), consistent with a previous study under experimental conditions.[22] Thus, we can conclude that the additional intermediate focal point in the trifocal lens was effective, and it did not seem to negatively impact distance or near vision.

Spectacle independence in our meta-analysis did not result in significant improvement in trifocal IOLs. There are two probable reasons for this outcome: first, spectacle independence in bifocal IOLs was already very high (ceiling effect), both in our meta-analysis (mean value of 71%) and in a previous meta-analysis (with a mean value of 80.1% reported by 63 studies)[7]; second, the number of included studies was still too limited (only two). To further compare the spectacle independence between them, more studies are necessary, especially studies reporting spectacle use at the intermediate distance specifically. [56] Before then, it is too early to conclude that trifocal IOLs could achieve spectacle independence more frequently.

Patient satisfaction is, without a doubt, a very significant issue to directly decide which multifocal IOL option becomes more prevalent.[1,57,58] In our meta-analysis, no significant difference in satisfaction was observed between IOLs. However, only two studies made direct comparisons regarding patient satisfaction. The limited number could be a reason that we did not obtain significant results. Another reason could be that patient satisfaction for bifocal IOLs was already very good (ceiling effect), both in our results (93% to 100%) and in a previous meta-analysis (61.8% to 100%).[7] Patient satisfaction is a very complex issue, and many aspects, in addition to the technological aspects of IOLs, such as characteristics of patients and the role of the surgeon, could affect patient satisfaction. [55,59,60] Thus, it would not to be an easy task to compare patient satisfaction between different IOLs.

Contrast sensitivity is a test required by the Food and Drug Administration (FDA) in evaluating multifocal IOLs, [58] and the FDA has placed a warning label on the ReSTOR and ReZoom IOLs for exercising caution when driving at night or in other poor visibility conditions because of the decrease in contrast sensitivity. Decrease in contrast sensitivity is a well-known side effect of bifocal IOLs.[47,61] Further, bifocal IOLs can cause up to a 50% reduction in contrast sensitivity, [62] with two thirds of 31 studies in a previous meta-analysis[7] reporting reduced contrast sensitivity in bifocal IOLs, compared with monofocal IOLs. As in our meta-analysis, under photoptic conditions (with luminance of 85 cd/m^2^), the trifocal IOL group had significantly better performance at 3 cpd in Mojzis et al.’s study[51]. In the meanwhile, all trifocal IOL groups had better performance at 6 cpd and 12 cpd, but the difference is not statistically significant. However, under mesoptic (with luminance of 3 cd/m^2^) conditions, performance in the bifocal group was statistically significantly better at 6 cpd (P <0.01) in Jonker et al.’s study.[48] In Plaza et al.’s study, [47] also under mesoptic conditions, no significant differences were observed between the trifocal and bifocal groups at frequencies of 1.5, 3.0, 6.0 and 12.0 cpd. More studies are needed to compare contrast sensitivity performance between trifocal and bifocal IOLs, especially under mesoptic conditions.

Only two studies in our meta-analysis made direct comparisons regarding the occurrence of photic phenomena (mainly include glare and halo). Both the trifocal and bifocal groups suffered halo and glare. In Carson et al.’s study, [20] halos surrounding the simulated headlight targets were smaller with the bifocal IOL compared with the trifocal IOLs, and trifocal IOLs were associated with increased background halos. Photic phenomena were usually reported by a validated questionnaire, such as the NEI-RQL, [48]NEI VFQ[49,52], and VF-14[50]; a varied methodology would make it inconvenient to directly compare photic phenomenon rates or various postoperative follow-up times (follow-up times varied in our study from 3 m to 12 m). The longer that the follow-up term was, the fewer that the number of patients was complaining about halos and glare, and patients have a tendency to become more tolerant of photic phenomenon over approximately 6 months [7]. We did not get the conclusive results about which type of IOL that has better performance in photic phenomenon. Further studies using quantitative instruments, such as halometry[63] or light distortion analysis[64], are needed.

Residual sphere and spherical equivalent (SE) did not show significant differences between the trifocal and bifocal groups, but residual cylinder was significantly lower in the trifocal group. However, we found that preoperative cylinder was significantly lower in the trifocal IOL group in Gundersen et al.’s study [49] and Plaza et al.’s study (AT LISA tri 839MP IOL subgroup), [46] and it was insignificantly lower in the trifocal group in two studies.[33] At the same time, two studies failed to mention the precondition of the cylinder.[50,52] Only one study reported an insignificantly lower preoperative cylinder in the trifocal group.[48] It is possible that the surgeon chose patients with less preoperative cylinder to be trifocal candidates, especially in a non- randomized study.

Serious postoperative complications were rare, with most studies reporting no adverse events regardless of IOL type; decentration was the only complication found in our meta-analysis. Current studies have not routinely included adverse complications in their outcome measurements [7]; thus, a larger population study with routine measurement of postoperative complications is needed.

Inevitably, the present meta-analysis had several limitations. First, publication biases can occur; to minimize them, we conducted an electronic search and a manual search of the references of the relevant studies to identify all of the potential relevant articles, while we excluded special reports and unpublished data, which can cause publication bias. Second, it should be noted that not all of the trials were randomized treatments, which can be explained by certain ethical constraints.[5] Even for RCTs, studies have seldom had adequate sequence generation or adequate methods to achieve allocation concealment, and none of the studies mentioned masking. RCTs that evaluate the visual outcomes after implantation of multifocal IOLs are still very rare; thus, more high-quality RCTs, multicenter RCTs in particular, are expected. Third, one study received grants from Alcon Laboratories and Carl Zeiss Meditec, [48] one study was funded by FineVision, [49] and one study received grants from Alcon Laboratories. [52]

## Conclusions

In this meta-analysis, the results demonstrated the efficacy of using a trifocal approach, compared with the bifocal IOL. In the aspect of intermediate visual acuity performance, trifocal IOL technology (especially the AT LISA tri 839MP IOL) had a clear advantage over the bifocal IOL, while both of them had excellent performance in distance visual acuity. Near visual acuity was not negatively impacted by trifocal IOLs compared with bifocal IOLs and could actually be positively enhanced by the AT Lisa trifocal 839M trifocal. However, more evidence is needed to decide which type of IOL has better performance in spectacle independence, patient satisfaction rate and photic phenomena. Postoperative complications following implantation both of trifocal and bifocal IOLs are rare and are often amenable to treatment.

## Supporting information

S1 Fig Quality of evidence: UDVA. (JPEG)

S2 Fig Risk of bias graph: Review authors' judgments of each risk of bias item presented as percentages across all of the included studies. (PDF)

S3 Fig Quality of evidence: UIVA. (JPEG)

S4 Fig Quality of evidence: UNVA. (JPEG)

S5 Fig Quality of evidence: Spectacle independence, satisfaction and complications. (JPEG)

S6 Fig Quality of evidence: Residual sphere and sphere equivalent. (JPEG)

S7 Fig Quality of evidence: Residual cylinder. (JPEG)

S1 File Table PRISMA 2009 checklist. (DOC)

S2 File EDITORIAL CERTIFICATE. (PDF)
