# Supplementary figures and images for: Comparison of clinical performance between trifocal and bifocal intraocular lenses: A meta-analysis
Source: PLoS One. 2017 Oct 26;12(10):e0186522. doi: 10.1371/journal.pone.0186522 (PMC5657996; doi:10.1371/journal.pone.0186522)

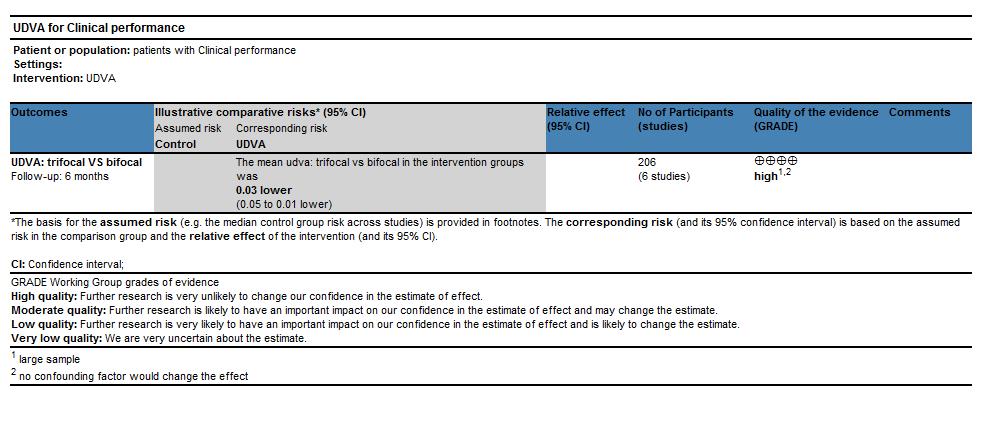

Supplement: S1 Fig — (JPEG) [file pone.0186522.s001.jpeg]

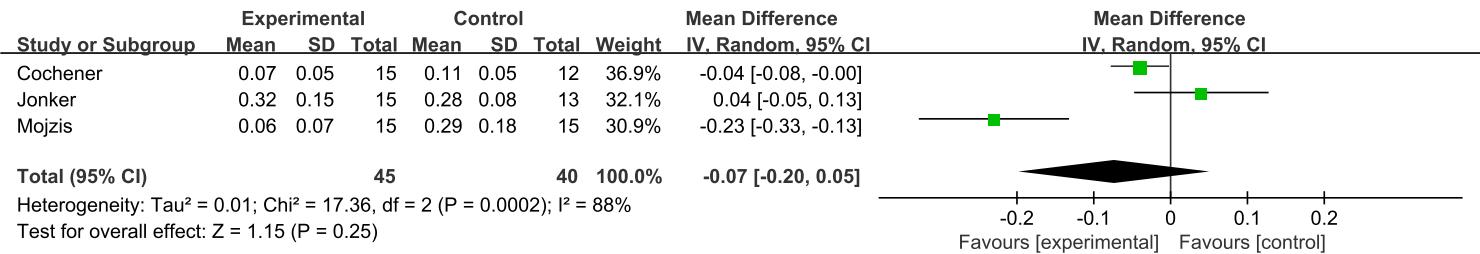

Supplement: S2 Fig — (PDF) [file pone.0186522.s002.pdf]

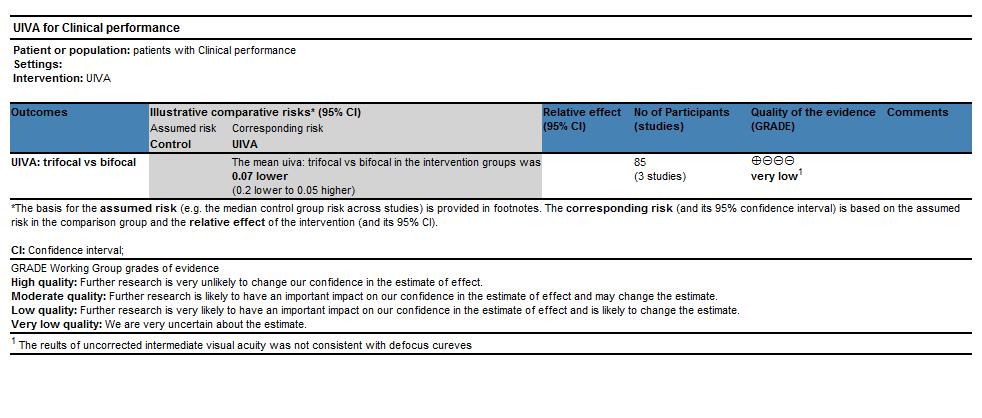

Supplement: S3 Fig — (JPEG) [file pone.0186522.s003.jpeg]

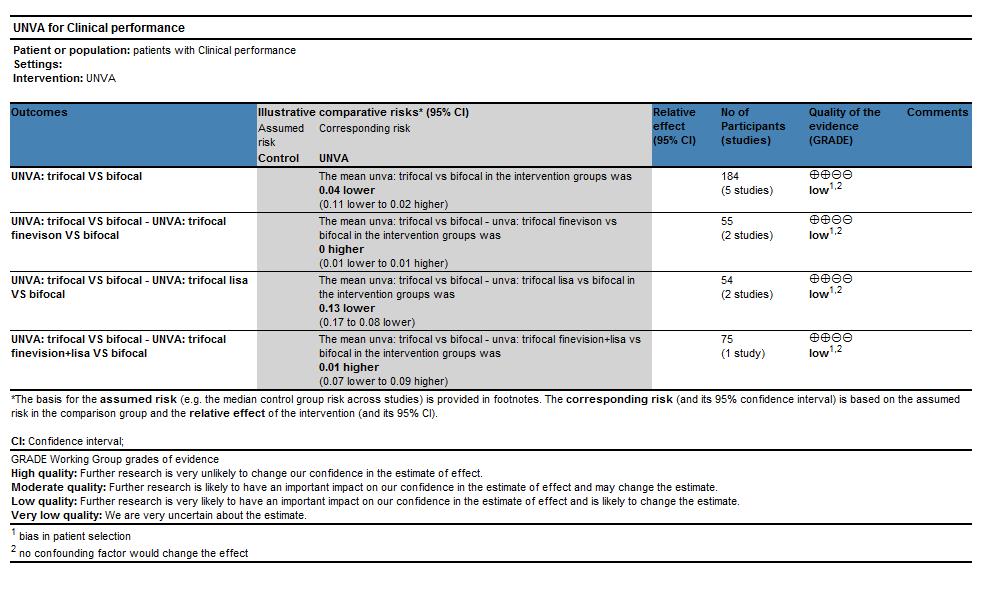

Supplement: S4 Fig — (JPEG) [file pone.0186522.s004.jpeg]

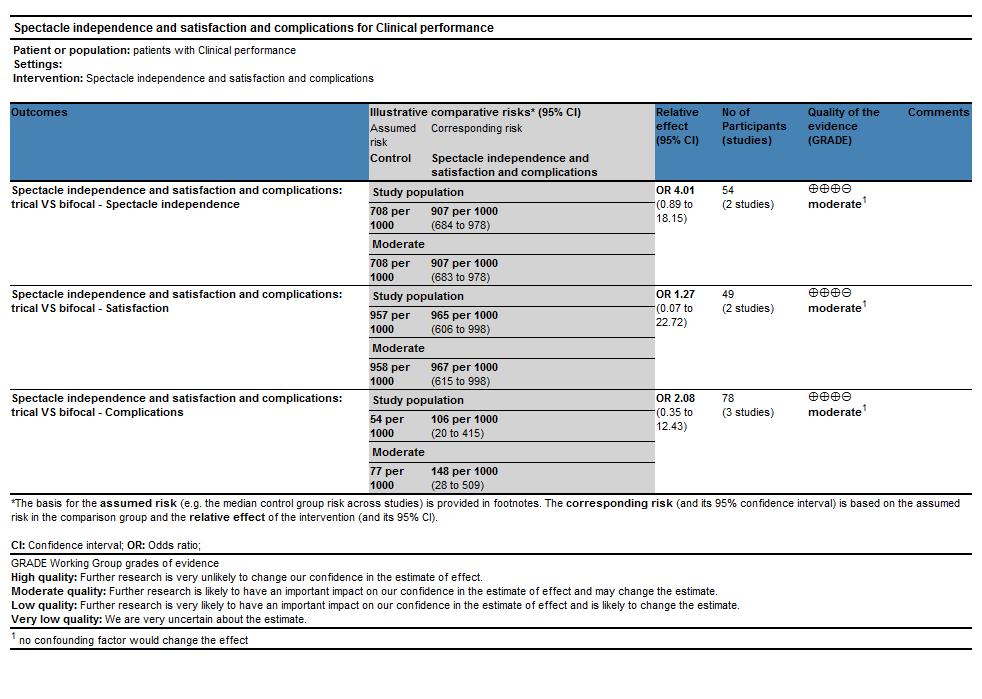

Supplement: S5 Fig — (JPEG) [file pone.0186522.s005.jpeg]

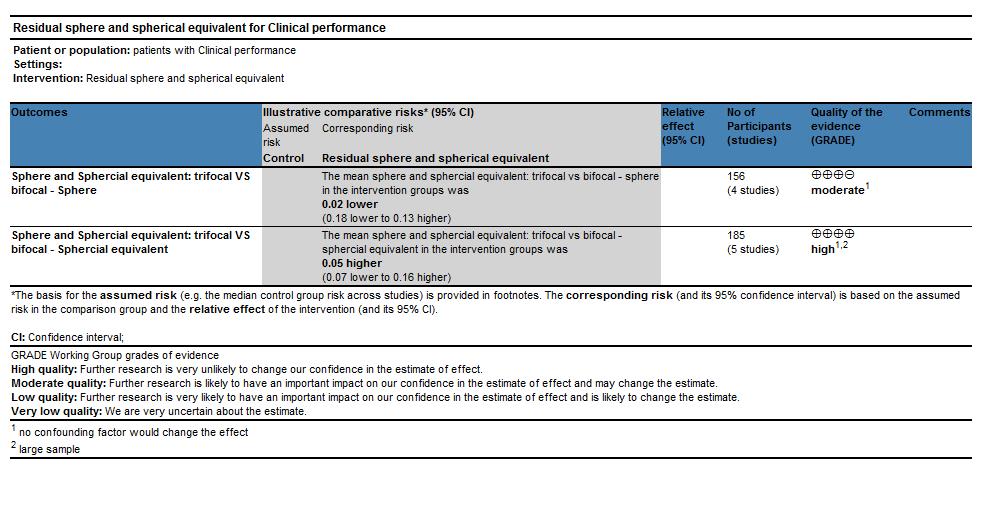

Supplement: S6 Fig — (JPEG) [file pone.0186522.s006.jpeg]

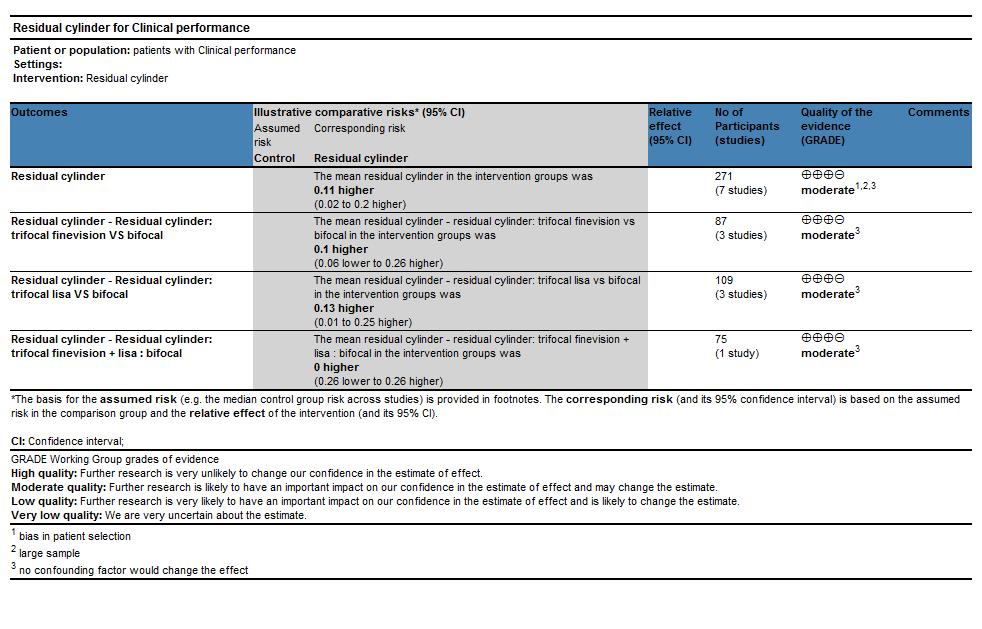

Supplement: S7 Fig — (JPEG) [file pone.0186522.s007.jpeg]
